# Using e-learning to advance advocacy and leadership in supply chain management

**DOI:** 10.1186/2052-3211-7-S1-O17

**Published:** 2014-12-17

**Authors:** Carole Piriou, Manusika Rai, Griet Samyn

**Affiliations:** 1 i+solutions, Woerden, Netherlands

## Background

Higher education programmes preparing health system managers for their jobs seldom include supply chain management (SCM) in the curriculum. However, SCM knowledge and skills are necessary not only for health workers, but also for managers and policy makers. Indeed, SCM awareness at decision-making level is essential to facilitate the establishment of policy frameworks giving SCM a place within health systems priorities and enabling the allocation of sufficient resources for SCM staffing and operations.

## Method

i+solutions, in collaboration with the Swiss Tropical Institute of Public Health, developed an e-course on the Introduction to SCM in healthcare as part of a MBA in International Health Management. The course was offered on a comprehensive learning platform, based on a constructivist educational approach, where participants learn from each other and their experiences, in addition to basic theory. Formal academic assessment tools, academic assignments and students’ feedback were used to evaluate the outcome of the course.

## Results

Participants came from global/public health backgrounds, development cooperation or other related fields with a majority without any previous knowledge of SCM. All participants successfully passed the final exam. The feedback on the pedagogic approach and the course content was positive. Furthermore, participants have contributed to the continual improvement of the course content by providing their varied perspectives, while learning from each other through interactions on discussion platforms. In addition to general SCM topics, the course helped appreciate people’s participation in healthcare systems and the significance of taking cultural perspectives into account.

## Discussion

Future leaders in global health can be equipped with a basic understanding of SCM through affordable, low-impact interventions such as e-learning modules. While it remains to be seen whether this translates into SCM decision-making in their professional lives. Larger number of students would need to be engaged in such training in order to push SCM higher on the global health agenda and integrate in thoroughly in health systems design. IT based learning platforms have a distinct advantage over traditional teaching in that, even after training is completed, participants can continue to interact and exchange successes and challenges.

## Lessons learned

Background concepts such as essential medicines, task shifting, and donor-funded health financing have to be defined and addressed in order to identify SCM challenges in a context understandable by all. Social learning has to be promoted to stimulate post-course online involvement and facilitate the assessment of the course impact in the field.

